# Assessing nonlinearities and heterogeneity in debt sustainability analysis: a panel spline approach

**DOI:** 10.1007/s00181-022-02284-8

**Published:** 2022-08-12

**Authors:** Benjamin Owusu, Bettina Bökemeier, Alfred Greiner

**Affiliations:** Department of Business Administration and Economics, Bielefeld University, P.O. Box 100131, 33501 Bielefeld, Germany

**Keywords:** Debt sustainability, Fiscal response function, Panel spline estimation, H63, E62

## Abstract

This paper empirically studies public debt sustainability with the penalized panel splines approach for 25 EU economies from 2000 to 2019 by estimating the response of the primary surplus to lagged debt relative to GDP, respectively. A positive coefficient on average indicates sustainable policies, which is supported by all our results. Moreover, we show that this relationship is not homogeneous across the distribution of the debt ratios but varies with the magnitude of public debt to GDP. The estimations reveal a strongly increasing reaction for small and high debt ratios while it is rather flat for intermediate levels. This holds for normal times, too, whereas during years of economic crisis a monotonously increasing response can be observed. Additionally, for a cluster consisting of smaller EU economies, there is an indication of ‘fiscal fatigue’, meaning that the effort of active fiscal counter-steering peters out for high ratios of public debt to GDP. The same effect can be observed for the whole sample and a sample including the large EU economies, once Greece is removed.

## Introduction

With the 2008/2010 European debt crisis, the interest in public debt sustainability analyses has revived. Further, the current Covid-19 pandemic affecting countries around the world forces governments to react in order to protect the societies and to cushion the negative economic effects with the help of huge rescue and recovery programs that are mainly financed by issuing bonds. Thus, these policies affect both the current and the future budget as well. Therefore, studying public debt sustainability and fiscal discretionary policies is as urgent as ever.

Based on the seminal contributions by Bohn (1995, 1998), we assess public debt sustainability by analyzing the reaction of the primary balance to changes in public debt relative to the gross domestic product (GDP), respectively. If a government reacts to a rise in the public debt ratio by actively adjusting its discretionary fiscal policy in terms of higher primary surpluses, the debt policy is considered to be sustainable. Usually, the relationship is empirically tested in a single equation linear regression model. However, standard econometric specifications, assuming linearity between a response and explanatory variable, could lead to model misspecification if the true data generating process is nonlinear and hence, could lead to wrong inferences. Therefore, it seems necessary to resort to nonparametric or semi-parametric models.

The need to increase the size of the data sets to improve statistical inference and to study the dynamic relationships between variables has made panel studies popular in applied macroeconomics research. The basic specification in panel studies is the fixed effects model, where the unobserved time-invariant individual heterogeneities are allowed to correlate with the regressors. That model assumes a linear relationship between the response variable and the covariates and allows to measure the marginal effects of the latter on the former.

However, in this paper, we intend to get additional insight into the data generating process and go beyond the standard linear fixed effects model that is commonly used in panel debt sustainability analyses. Thus, we question the hypothesis that the response of the discretionary fiscal policy is uniform across the distribution of the debt ratios, but, we allow it to vary with the magnitude of the debt ratios. (“Does size matter?”) Particularly, we can show that the reaction of the structural primary balance changes with the size of the debt ratio, implying that in situations with low debt ratios the response coefficient is expected to be different as compared to situations with high debt ratios. In addition, we consider two different clusters of EU economies to allow for country specifics and we distinguish between times of economic crisis and normal times, where we use AMECO data for 25 EU economies from 2000 to 2019. The reasoning for focusing on this sample is obvious against the background of the sovereign debt crisis in Europe around the years 2010. The estimations demonstrate that the reaction to higher debt ratios differs in the two clusters and that it is higher in times of crisis than in normal times. Thus, our estimations provide support for the hypothesis of heterogeneity in the data. These refinements are particularly important for policy implications and help to improve recommendations as they indicate that the status of the current debt and the economic situation is essential for the assessment and evaluation of fiscal sustainability. Our results show that, yes, size does matter! The level of the explanatory variable influences the reaction coefficient and, thus, sustainability. This means policy recommendations need to consider the status of the current debt situation in order to be successful, as the reaction shows different behavior for low debt levels compared to medium or high ratios.

The contribution of the paper is threefold; firstly, we applied a semi-parametric fixed effects model (Panel penalized splines) to study the nonlinearities in debt sustainability analysis for EU countries. Hitherto, only limited numbers of papers had explored nonlinear models for debt sustainability analysis in the panel data context. The panel penalized spline models provide the opportunity to visualize the behaviour of the reaction coefficient (primary surplus) over the distribution of the covariate of interest (lagged debt-ratio). Secondly, the paper employs a clustering algorithm to segregate our datasets due to the potential heterogeneity of the countries in the panel. This enabled us to study the debt sustainability characteristics of each cluster in the EU. Finally, the paper adds to the empirical literature of nonlinear models in debt sustainability analysis.

However, there are still limitations to the study. Our data set covers observations until 2019 only. Certainly, the effects of the pandemic requiring immediate unprecedented policy responses by governments and leading debt ratios to skyrocketing levels are not covered yet. Also, technically the applied models do not take cross-sectional dependence into account. All these issues are open for further research.

The rest of this paper is organized as follows. The next section presents a short literature review. Section 3 briefly discusses the theoretical background and Sect. 4 presents the estimation method and the outcomes. Finally, Sect. 5 summarizes the main results, indicates further research and concludes. Additional information about the empirics can be found in “Appendix”.

## Literature overview

Statistical testing of whether the inter-temporal budget constraint of a government is fulfilled began with the paper by Hamilton and Flavin (1986). Those authors tested whether the present-value borrowing constraint of the US federal government holds with annual data from 1960 to 1984. They did so by analyzing if the time series of the US federal public debt contains a bubble term that would indicate that public debt exceeded the present value of expected future primary surpluses, implying an unsustainable debt policy. Hamilton and Flavin performed several tests and found evidence for the sustainability of the US federal debt policy.

The test procedure applied by Hamilton and Flavin has been criticized by Wilcox (1989) because their test does not allow for a stochastic interest rate. Therefore, he proposed a different test where the discounted time series of public debt should be analyzed and if that series converges to zero, sustainability of the public debt would be given. Applying that test to the same time series Hamilton and Flavin used, Wilcox finds evidence that the US federal debt is unsustainable.

The result obtained by applying the test proposed by Wilcox heavily depends on the interest rate with which the series of public debt is discounted. Since this is a random variable and realizations of that variable in the past do not give information about future interest rates, it has been argued that tests should be resorted to that yield outcomes which are independent of the interest rate. Hakkio and Rush (1991), for example, tested for the cointegration of revenues and expenditures. The idea behind that approach is that a cointegrating relation between spending and revenues implies a stationary first difference and, thus, a sustainable debt policy for a positive interest rate.

Another test that does not rely on the interest rate is the one proposed by Trehan and Walsh (1991). They test whether public deficits inclusive of interest payments grow at most linearly. If that property is fulfilled a given series of public debt is sustainable because any time series that grows linearly converges to zero if it is exponentially discounted, provided the real interest rate is positive. Denoting by *B* public debt and by *r* the interest rate, another test proposed by Trehan and Walsh (1991) is to analyze whether a quasi-difference of public debt, $B_t-\lambda B_{t-1}$ with $0\le \lambda <1+r$, is stationary and whether public debt and primary surpluses are cointegrated. If government debt is quasi-difference stationary and public debt and primary surpluses are cointegrated, public debt is sustainable.

The dependence of sustainability tests on the interest rate has been heavily criticized by Bohn (1995, 1998), too, since future realizations of that random variable are unknown. Therefore, he tested whether the primary surplus relative to GDP is a positive function of the debt to GDP ratio that rises at least linearly with higher debt to GDP ratios. The intuition behind that procedure is that a positive reaction of the primary surplus to higher debt relative to GDP, respectively, implies mean reversion of the debt to GDP ratio and a stationary debt to GDP ratio is sustainable in a growing economy. This test is very plausible because it has a nice economic intuition: if governments run into debt today they have to take corrective actions in the future by increasing the primary surplus. Otherwise, public debt will not be sustainable. Many applications of such a fiscal response function as suggested by Bohn have followed. A nice recent overview can be found for instance in Beqiraj et al. (2018). Recently, some applications have focused on nonlinear fiscal behaviour and studied “fiscal fatigue”, which is a reverse in behavior of the primary balance as debt ratios become very high, the response peters out and becomes negative. This has been introduced by Ghosh et al. (2013) and studied by Checherita-Westphal and Zdarek (2017) as well as Fournier and Fall (2015) for example. Table 1 below provides a summary of papers regarding European debt sustainability in the panel data context.

**Table 1:** Summary of panel fiscal sustainability research in the EU context

Authors	Sustainability test	Period and country	Findings
Afonso and Rault (2010)	Stationarity of debt and cointegration between revenue and expenditure	15 Selected EU countries (1970–2006)	Fiscal stance sustainability confirmed
Baldi and Staehr (2016)	Fiscal reaction function of primary balance, debt and business cycle viables	Different groups of EU countries (2001–2004)	Sustainable fiscal stance for all groups post financial crisis
Checherita-Westphal and Zdarek (2017)	Fiscal reaction function of primary balance response to debt	18 Euro Area countries (1970–2013)	Sustainable fiscal stance
Claeys (2007)	Cointegration between revenue, spending and net interest payment	Selected European countries (1970–2001)	Sustainable fiscal policy
Lee et al. (2018)	Fiscal reaction function of primary balance response to debt	EU regional groups (1950–2014)	Varied results depending on the region
Medeiros (2012)	Fiscal reaction function and VAR	15 EU Member States	Fiscal stance sustainability in the weaker sense
Prohl and Schneider (2006)	Cointegration between budget deficit and public debt	15 EU countries (1970–2004)	Fiscal stance sustainability confirmed
Prohl and Westerlund (2009)	Cointegration between revenue and expenditure	15 European countries (1970–2004)	Sustainable fiscal policy
Schalk (2012)	Fiscal reaction function	12 Eurozone Countries (1999–2010)	Varied fiscal response depending on the quantile
Stoian et al. (2022)	Fiscal reaction function	26 European Union countries (2005–2018)	Varied fiscal response depending on the quantile

## Theoretical background

To get a deeper insight into the logic of the test proposed by Bohn (1995), Bohn (1998) and the fiscal response function, we consider the accounting identity describing the accumulation of public debt in continuous time that is given by the following differential equation:

1 $$\begin{aligned} \dot{B}(t)=r(t)B(t)-S(t), \end{aligned}$$

with $B(t)>0$ real public debt at time *t*, $r(t)>0$ the real interest rate, $S(t)\in \text{[ }0.8mm][l]{\mathrm{I}}\mathrm{R}$ the real government surplus exclusive of interest payments and the dot over a variable stands for the derivative with respect to time $\mathrm{d}/\mathrm{d}t$. A government is said to follow a sustainable debt policy if its inter-temporal budget constraint is fulfilled, i.e. if the present value of public debt converges to zero asymptotically. The latter means that $\lim _{t\rightarrow \infty }e^{-C_1(t)} B(t)=0$ holds, with $C_1(t)={\int _{t_0}^{t}r(\mu )\mathrm{d}\mu }$ the discount rate.

Assuming that the government in the economy determines the primary surplus to GDP ratio, $s(t)=S(t)/Y(t),$ such that it is a positive linear function of the debt to GDP ratio, $b(t)=B(t)/Y(t),$ and of a term that is independent of public debt, $\phi (t)$ (see Bohn 1995, 1998; Greiner and Fincke 2015; Afonso and Jalles 2019), the primary surplus ratio can be written as

2 $$\begin{aligned} s(t)={\psi }\, b(t) +\phi (t), \end{aligned}$$

where $\psi$ is the reaction or response coefficient determining the change of the primary surplus to variation in public debt, relative to GDP, respectively. The parameter $\phi (t)\in \text{[ }0.8mm][l]{\mathrm{I}}\mathrm{R}$ is affected by other economic variables, such as social spending or transitory government expenditures in general. As regards $\phi (t)$ we posit that it is bounded from above and from below by a certain finite number that is constant over time. We should also like to emphasize that $\phi (t)$ cannot be completely controlled by the government. The government can influence that coefficient to a certain degree but it has not complete control over it because $\phi (t)$ is also affected by the business cycle for example that can affect temporary government outlays.

Using (2) the period budget constraint of the government (1) is obtained as

3 $$\begin{aligned} \dot{B}(t)=\left( r(t)-{\psi }\right) B(t)-\phi (t)\, Y(t). \end{aligned}$$

Integrating equation (3) and multiplying both sides by $e^{-C_1(t)}$ leads to

4 $$\begin{aligned} e^{-C_1(t)}B(t) = e^{-C_3(t)}\,B(t_0)-e^{-C_3(t)}\,\int _{t_0}^te^{-C_1(\mu )+C_2(\mu )+C_3(\mu )}Y(t_0)\phi (\mu )\mathrm{d}\mu , \end{aligned}$$

with *g*(*t*) the growth rate of GDP and $C_1(\mu )={\int _{t_0}^{\mu }r(\nu )d\nu }$, $C_2(\mu )=\int _{t_0}^{\mu }g(\nu )d\nu$, $C_3(\mu )=\psi \mu$. As regards the interest rate, we posit that the interest rate on government bonds exceeds the growth rate of GDP on average, $\int r(\mu )\mathrm{d}\mu >\int g(\mu )\mathrm{d}\mu$. We make this assumption because otherwise the inter-temporal budget constraint would not pose a problem for the government since it can grow out of debt in that case.

Now, assume that $\psi$ is positive on average so that $\lim _{t\rightarrow \infty }C_3(t)=\infty$ holds. Then, we get $\lim _{t\rightarrow \infty }e^{-C_3(t)}\, B(t_0)=0$ and the first term in equation (4) converges to zero.

Since $|\phi (t)|<\infty$ we can set $\phi (t) Y(t_0)=1$ so that it is sufficient to consider for the second term on the right hand side in (4) the following expression,

$$\begin{aligned} \Omega (t)=\frac{\int _{t_0}^t e^{-C_1(\mu )+C_2(\mu )+C_3(\mu )}\mathrm{d}\mu }{e^{C_3(t)}}\, . \end{aligned}$$

If $\int _0^{\infty } e^{-C_1(\mu )+C_2(\mu )+C_3(\mu )}\mathrm{d}\mu$ remains bounded $\lim _{t\rightarrow \infty }C_3(t)=\infty$ guarantees that $\Omega$ converges to zero. In case of $\lim _{t\rightarrow \infty }\int _{t_0}^{t} e^{-C_1(\mu )+C_2(\mu )+C_3(\mu )}$ $\mathrm{d}\mu =\infty$, the limit of $\Omega$ is obtained by applying l’Hôpital as

$$\begin{aligned} \lim _{t\rightarrow \infty }\Omega (t)=\lim _{t\rightarrow \infty }\frac{e^{-C_1(t)+C_2(t)}}{\psi }. \end{aligned}$$

These considerations demonstrate that a strictly positive reaction coefficient on average $\psi >0$ and a positive interest rate—growth rate gap $\lim _{t\rightarrow \infty } \int r(\mu )\mathrm{d}\mu -\int g(\mu )\mathrm{d}\mu = \infty$ must hold to imply that the debt policy of a government is sustainable (see Greiner and Fincke 2015, chap. 2.2-2.5 for details). The latter requirement means that the interest rate/growth rate difference must be positive on average in order for the present value of public debt to converge to zero asymptotically. It must be pointed out that the reaction coefficient $\psi$ can be varying and it may even be negative for some time periods. However, on average that coefficient must be positive. The aspect regarding the interest rate—growth rate interval becomes particularly important when considering ratios to GDP. A shortcoming of the former analysis is that it implicitly assumes that the primary surplus can grow without upper bound. However, a positive but small reaction coefficient on average does not necessarily guarantee a bounded debt to GDP ratio. It can be demonstrated that the public debt to GDP ratio *b*(*t*) remains bounded if the reaction coefficient $\psi >\int _{t_0}^t\left( r(\mu )-g(\mu )\right) \mathrm{d}\mu ,$ while it diverges to infinity in the case of $\psi \le \int _{t_0}^t\left( r(\mu )-g(\mu )\right) \mathrm{d}\mu .$ Thus, the reaction coefficient must exceed the difference between the interest rate on public debt and the GDP growth rate on average such that the debt to GDP ratio remains bounded.

Here, we should like to stress three aspects. Firstly, the previous calculations show the significance of the difference $\int r(\mu )\mathrm{d}\mu -\int g(\mu )\mathrm{d}\mu$. If the GDP growth rate exceeds the interest rate on public debt, the debt to GDP ratio remains bounded. In that case, the government can grow out of debt as already mentioned above. Second, a positive reaction coefficient that falls short of the difference between the interest rate and the GDP growth rate implies a rising debt to GDP ratio, if the interest rate exceeds the GDP growth rate. But, such a policy is not sustainable because it would require permanently rising primary surplus to GDP ratios which is not feasible because the primary surplus relative to GDP is bounded from above because the primary surplus can never exceed GDP. Thus, there exists a critical threshold value of the debt ratio beyond which sustainability is excluded. In that case the outstanding debt ratio is too large to be covered by the sum of discounted future primary surpluses (cf. Greiner and Fincke 2015, chap. 2.1, Prop. 3).

Third, and to finish our theoretical considerations, we want to point out that the reaction coefficient must not be constant but may be varying. A constant reaction coefficient is rather implausible. In fact, times series analyses provide strong empirical evidence that this coefficient is not constant for instance over time (see Greiner and Fincke 2015, chap. 2.2–2.5). We emphasize this point because in our empirical part we estimate a nonlinear model describing the relationship between the primary surplus and public debt. More concretely, our model is a semi-parametric model and the estimation outcome consists of the average coefficient and of a smooth function such that the actual coefficient for each observation consists of the average value plus the nonlinear part given in the figure. In particular, we allow the reaction coefficient to vary across the distribution of the debt ratio. We estimate the fiscal policy reaction in a flexible manner along the evolution of the debt ratio. See Greiner and Fincke (2015) for detailed proof regarding the above theoretical discussions.

The use of a nonlinear model is of interest because the question arises of which factors are responsible for variations in the coefficients. When analyzing the response of the primary surplus to variations in public debt, there is evidence that the reaction depends on the magnitude of the public debt ratio. For example, Ghosh et al. (2013) have analyzed 23 advanced economies over the period 1970–2007 and found that the reaction decreases once a critical value has been passed, what they refer to as ’fiscal fatigue’. Checherita-Westphal and Zdarek (2017) also explored ‘fiscal fatigue’ in euro-area economies with a focus on the primary balance.

## Empirics

### Estimation method

Regarding the methodology for the empirical estimation, we resort to the penalized splines fixed effects estimator according to Puetz and Kneib (2018). Such additive semi-parametric models have become increasingly popular in empirical works (Baltagi and Li 2002). We argue that this approach is desirable because nonparametric modeling does not impose restrictions regarding the functional relationship between the response variables and the regressors. Rather the functional shape of the co-variate effect is derived from the datasets. Pioneering works on penalized splines can be traced back to Hastie and Tibshirani (1990) with their introduction of generalized additive models which provide a flexible way of modeling the response of a dependent variate to co-variates. Subsequent contributions were made by Wood (2000) who introduced the mixed model representation of penalized splines, as well as Ruppert et al. (2003), Eilers and Marx (1996) and Kauermann (2005).

In panel data settings, the correlation between covariates and the unobserved time-invariant factors in the error term is prevalent. Baltagi and Li (2002), Su and Ullah (2006) and Henderson et al. (2008) have all proposed alternative semi-parametric fixed effects estimators where the nonlinearity is modeled via a kernel estimator. However, Puetz and Kneib (2018) argued that modeling fixed effects and nonlinearity with the Kernel estimator is computationally demanding especially with large datasets. Penalized splines on the other hand provide a flexible and convenient way of modeling the nonlinearity without complications and the fixed effects in the model can be conveniently handled by way of a first difference operator.

Following Puetz and Kneib (2018), we consider the following panel additive model

5 $$\begin{aligned} y_{i,t} = \mu _{i} + \sum _{g=1}^{p} f_{g}(x_{gi,t}) + v_{i,t} \end{aligned}$$

where $i = 1,\ldots ,N$ represent the individual countries and $t=1,\ldots T$ represent the time period, $y_{i,t}$ is the response variable, whilst $\mu _{i}$ accounts for the time invariant unobserved heterogeneity also known as the individual fixed effects in this case. By this, we allow the unobserved heterogeneity to correlate with the regressors instead of the error term. The variable $v_{i,t}$ represents the error term which is assumed to be normally distributed with zero mean and a constant variance. The functions $f_{1}(x_{gi,t})$,..., $f_{p}(x_{gi,t})$ are the nonlinear effects of the *p* continuous co-variates which can be approximated by a penalized B-splines according to Eilers and Marx (1996) together with a penalty term applied to penalized least squares criterion. The aim of the penalty term is to penalize too much variability in the model as a way of regularizing the estimation in order to deal with overfitting to the data.

Assuming that (5) holds for each point, we could express the lag as

6 $$\begin{aligned} y_{i,t-1} = \mu _{i} + \sum _{g=1}^{p} f_{g}(x_{gi,t-1}) + v_{i,t-1} \end{aligned}$$

in order to eliminate the fixed effects, we subtract (6) from (5) to obtain

7 $$\begin{aligned} \Delta y_{i,t} = \sum _{g=1}^{p} [f_{g}(x_{gi,t}) - f_{g}(x_{gi,t-1})] + v_{i,t} -v_{i,t-1} \end{aligned}$$

where $\Delta$ is the difference operator. The nonlinear function $f_{g}$ is approximated by the weighted sum of $d_{g}$ B-spline basis functions $B_{g1},\ldots ,B_{gd}$ such that

8 $$\begin{aligned} f_{g}(x_{gi,t}) = \sum _{g=1}^{d_{g}}B_{gj}(x_{gi,t})\beta _{gi,t} = z_{g}^{T}(x_{gi,t})\beta _{g}. \end{aligned}$$

Inserting (8) into (7) and applying the difference operator once again, we have

9 $$\begin{aligned} \Delta y_{i,t} = \sum _{g=1}^{p}[\Delta z_{g}(x_{gi,t})]^{T}\beta _{g} + \Delta v_{i,t}. \end{aligned}$$

For the sake of simplicity, (9) can be written in compact matrix notation as

10 $$\begin{aligned} \Delta y = \sum _{g=1}^{p} \Delta z_{g}\beta _{g} + \Delta v \end{aligned}$$

where $\Delta y = (y_{12} - y_{11},\ldots , y_{N,T}-y_{N,T-1}^{T})$ is a column vector with dimension $N(T-1)$ and $\Delta z_{g}$ is derived by taking the difference between $z_{g}$ and its lags which is of dimension $N(T-1) X d_{h}$.

In order to obtain a first difference penalized spline estimator, we minimize the penalized least square criterion below

11 $$\begin{aligned} \left[ \Delta y - \sum _{g=1}^{p}(\Delta z_{g})\beta _{g}\right] ^{T} \left[ \Delta y - \sum _{g=1}^{p}(\Delta z_{g})\beta _{g}\right] + \sum _{g=1}^{p}\lambda _{g}\beta _{g}^{T}k_{g}\beta _{g} \end{aligned}$$

where the matrix *k* is introduced and assigned to each smooth function to penalize too much variability of the adjacent coefficient in the vector $\beta _{g}$. This prevents overfitting the data to the model. $\lambda _{g}$ is the smoothing parameter which is the weight placed on the penalty term in the minimization criterion in (11). In reality, the smoothing parameters are unknown ex-ante. However, they can be estimated via maximum likelihood estimation by making use of the mixed model representation of the penalized splines. To estimate the smooth function $f_{g}$, equation (5) is identified such that $\sum _{i=1}^{N}\sum _{t=1}^{T} f_{g}(x_{gi,t}) = z_{g}\beta _{g}=0$, for all $g=1,\ldots ,p$. In order to account for serial correlation in the residuals which could arise as a result of first difference transformation, a Generalized Least Square (GLS) approach is used where the differenced model matrix ($\Delta z_{g}$) and $\Delta y$ in (11) are multiplied by a block diagonal matrix.^1^ This ensures that the estimator is robust against serial correlation resulting from first differencing. Another appealing feature of the semi-parametric fixed effects estimator is the estimation of the derivative of the smooth function as well as the computation of simultaneous confidence bands for inferences. See Wiesenfarth et al. (2012) and Puetz and Kneib (2018) for details regarding the simultaneous confidence bands.

### Empirical results

In this paper, we model a fiscal reaction function in analogy to Bohn (1998) where the primary surplus is assumed to be a linear function of debt, relative to GDP, respectively, and of other macroeconomic variables which serve as control variables. In contrast to Bohn we do not posit that the response of the primary surplus to public debt is linear, but, we estimate a semi-parametric model of the form

12 $$\begin{aligned} S_{i,t} = \mu _{i} + f(X_{i,t}) + \gamma Z_{i,t}^{T}+ v_{i,t} \end{aligned}$$

where the $S_{i,t}$ is the response variable, $X_{i,t}$ represents the variables which enter the model nonlinearly and hence $f(X_{i,t})$ is the penalized function which is orthogonal to the linear part of the model. $Z_{i,t}$ are other variables which are modeled linearly, $\gamma$ measures the impact of the linear regressors on the response variable and $v_{i,t}$ is the uncorrelated error term assumed to be centered around zero with a constant variance. We shall apply (12) to test the fiscal reaction function in this paper.

As a first step, all variables are modeled nonparametrically, meaning that we do not impose any restrictions on the relationship between the response variables and the covariates. For instance, if the true relationship between any of the co-variates and the response variable is indeed linear, the spline model will impose a linear relationship via the effective degree of freedom of the co-variate in question.^2^ This is particularly advantageous since we thus avoid misspecification of the model that would result if apriori restrictions were imposed that are inconsistent with the true data generating process.

The source of the data used for the empirical study is the European commission AMECO (2021) website. Regarding the response variable, we use the cyclically adjusted primary balance of the governments.^3^ In general the primary balance is the fiscal balance of the government excluding interest payments. We focus on the cyclically adjusted balance because it is devoid of shocks or one-off fluctuations and because this is consistent with the EU fiscal framework (see Mourre et al. 2013). It is expressed relative to GDP and denoted by pbratio with positive (negative) values indicating surpluses (deficits). The explanatory variables include the lagged debt-ratio of the previous period $t-1$ which constitutes our main co-variate of interest. Following the Barro (1979) tax smoothing principle^4^, we use the business cycle variable (YVAR) and the public expenditure gap (GVAR) as control variables. YVAR which is also known as the output gap is computed as the deviation of output (GDP) from its long-term trend. Similarly, we compute the GVAR as the deviation of real government spending from its long-term trend. We use the HP filter to estimate the trend components of output and real spending. In order to capture the influence of international trade, we include the net export-ratio that is given by the difference between exports and imports as a ratio to GDP. In addition, we include Inflation in order to explore its influence on the primary balance as a proxy for monetary policy effects.^5^ A total of 25 EU countries constitutes the sample with the exception of Lithuania and Croatia due to unavailability or missing data. The sample period is from 2000 to 2019, hence a total of 500 observations in annual frequency were generated.

Before we present our estimation results we give a summary statistics of the variables in Table 2. It can be realized that the average primary balance is positive, although rather small, and that the two variables of interest, the primary balance ratio and the debt to GDP ratio, are left-skewed and right-skewed, respectively. A correlation matrix presented in Table 5 in “Appendix” depicts a positive relationship between the primary balance and lagged debt-ratio even though the correlation coefficient is not so strong. Inflation, GVAR and YVAR all have negative relationships with primary balance. We also show the behaviour of the primary balance and lagged debt-ratio in Fig. 5 in “Appendix” for selected countries. One can observe a sharp decline in the primary balance ratio during the 2009/2010 period for the bigger economies. The lagged debt-ratio is relatively flat and sturdy except for Greece, Spain, Portugal and Italy which record a slight upward trend after the year 2010.

**Table 2:** Summary statistics

Statistic	Pbratio	Lagged debt-ratio	GVAR	YVAR	Inflation	Net export-ratio
Mean	0.000107	0.585548	1.80E−11	− 3.40E−11	0.905804	0.022806
Median	0.002366	0.540408	− 0.136394	− 0.255106	0.938120	0.015596
Maximum	0.097171	1.862386	52.88208	96.16156	1.166769	0.360148
Minimum	− 0.276920	0.037655	− 36.70577	− 106.6082	0.441318	− 0.206930
SD	0.030345	0.343700	6.337278	16.20572	0.128713	0.087258
Skewness	− 1.700262	0.903345	1.808153	0.221095	− 1.067245	1.257040

The next table gives the outcomes of our estimations for the full sample, both for the linear and for the nonlinear model.^6^

**Table 3:** Estimation results—full sample

Variables	Response variable: Pbratio										
	Panel linear fixed effects			Panel penalized spline					GMM		MG
	1	2	3	4	5	6	7	8	9	10	11
*Parametric*											
Lagged debt-ratio	0.073***	0.077***	0.078***	0.049***	0.055***	0.042***	0.042***	0.024***	0.052***	0.015**	0.062***
	(0.017)	(0.017)	(0.017)	(0.007)	(0.007)	(0.007)	(0.007)	(0.007)	(0.013)	(0.006)	(0.019)
GVAR	− 0.002***	− 0.002***	− 0.002***	− 0.002***	− 0.002***	− 0.002***	− 0.002***	− 0.002***	− 0.001*	− 0.001**	− 1.012***
	(0.0001)	(0.0001)	(0.0001)	(0.000)	(0.000)	(0.000)	(0.000)	(0.000 )	(0.0006)	(0.000)	(0.005)
YVAR	− 0.0001	− 0.0001*	− 0.0001*	0.00001	0.00004	0.0001	0.0001	0.0001	0.0001	0.0004	0.0004
	(0.0001)	(0.0001)	(0.000)	(0.000)	(0.000)	(0.0001)	(0.000)	(0.000)	(0.000)	(0.000)	(0.0189)
Inflation		0.044	0.042		− 0.022**	− 0.050***	− 0.049***	− 0.043***	− 0.037**	− 0.014*	− 0.048*
		(0.035)	(0.035)		(0.009)	(0.009)	(0.009)	(0.009)	(0.015)	(0.008)	(0.028)
Net export-ratio			− 0.024			0.154***	0.181***	0.172***	0.073	0.044	0.015
			(0.039)			(0.029)	(0.030)	(0.029 )	(0.058)	(0.027)	(0.057)
Lagged pbratio									0.423***	0.599***	
									(0.042)	(0.041)	
*Nonparametric*											
edf(lagged debt-ratio)				4.856***	5.380***	5.102***	5.806***	6.987***			
edf (GVAR)							6.995***	6.986***			
edf (YVAR)							2.987**	3.399***			
edf (Net export-ratio)							6.360***	6.423***			
Adj $R^{2}$	0.292	0.293	0.292	0.382	0.379	0.409	0.609	0.574			0.910
Observ	475	475	475	475	475	475	475	475	450	925	500
Sargen test									20.0(0.9)	21.2(0.9)	
Autoc test (1)									− 2.4 (0.027)	− 2.5(0.01)	
Autoc test (2)									0.45 (0.650)	1.03( 0.310)	
Wald test of coefficients									231.4(0.000)	412.6(0.000)	

Standard error are indicated in parenthesis

Model 1 to 7 are the full sample specification whilst model 8 represents sample without Greece. Model 1 to 8 are based on estimations according to Puetz and Kneib (2018). Model 9 depicts results from the Arellano and Bond (1991) estimator whilst model 10 shows results from Blundell and Bond (1998) estimator. Model 11 presents results from Pesaran and Smith (1995) mean group estimator

*, ** and *** indicates statistical significance at 10  5 and 1  respectively

Table 3 presents the standard linear fixed effects results for models 1 through 3, with the latter giving the full model including all our control variables. It shows that the response coefficient is positive and statistically significant for all three parametric specifications, indicating sustainable debt policies. Additionally, public spending (GVAR) turns out to have a small negative influence on the structural primary balance, while the business cycle effect represented by YVAR is rather weak and significant only at the 10% level in models 2 and 3. The latter result may be due to the fact that we use the cyclically adjusted primary balance. Inflation and trade did not turn out to be statistically significant.

**Fig. 1:**
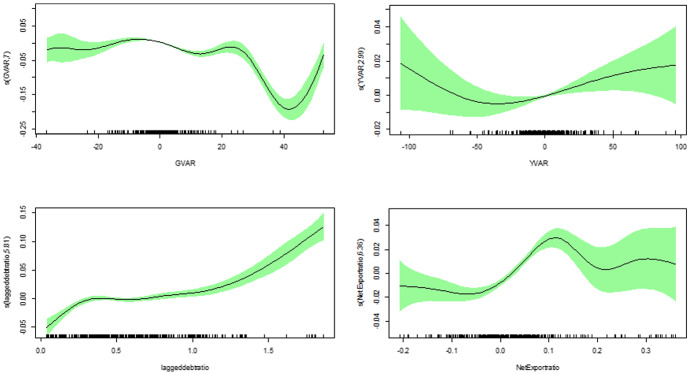
Full sample—smooth functions. Smooth functions of the nonparametric variables. Vertical axis depicts the estimated degree of freedom which governs the (non)linearity of the function

Next, we argue that allowing for a more flexible approach, where the effects are modeled in a nonparametric manner, turns out to be more informative. The second part of Table 3 with specifications 4 to 7 presents the results for the penalized spline model accounting for nonlinear effects of the lagged debt-ratio, GVAR, YVAR and of net export-ratio.^7^ Net export-ratio and Inflation are now statistically significantly positive and negative, respectively. The results for the lagged debt-ratio show a significantly positive average response that, however, is a little smaller compared to the outcome of the linear estimations. But the estimated degrees of freedom of the smooth term for the lagged debt-ratio with values around 5 indicate an alternating behavior across the distribution and is statistically significant. The same holds for the other variables, except Inflation. The shape of the smooth functions is depicted in Fig. 1.

Figure 1 shows that there are indeed variations in the effects of the variables across the distribution of the explanatory variables. It should be recalled that negative (positive) values in Fig. 1 imply that the actual parameter is below (above) the average and the actual coefficient just equals the estimated average plus the respective value given in the figure. For our variable of interest, the lagged debt-ratio, it can be realized that there is an increase in the reaction coefficient until the lagged debt-ratio reaches about 30 percent, followed by some flat part and again a distinctive increase once the debt to GDP ratios rise above 100 percent. This information from the nonlinear modeling yield additional insight compared to the estimated parameters obtained in the standard linear fixed effects model. It clearly reveals heterogeneity in the relationship.

There could be a potential correlation between GVAR and real government spending and also between YVAR and GDP since both GVAR and YVAR were obtained from government spending and GDP, respectively. Hence, potential endogeneity problems could arise since GDP and real government spending are part of the error term because they are not considered as part of the model. Since the panel splines do not directly deal with endogeneity, we therefore employ the Generalized Method of Moments estimator where we instrument lags of GDP and lags of real government spending which are both assumed to be orthogonal to the error term. We make use of the difference GMM estimator by Arellano and Bond (1991) and the system GMM estimator by Blundell and Bond (1998). Results obtained from both models are quantitatively comparable to the panel penalized splines. Results of GMM estimator can be found in Table 3 where specifications 9 represents the Arellano and Bond model and specification 10 is the results from the Blundell and Bond estimator. One lag of the primary balance ratio is added to the GMM model to account for potential autocorrelation that could emanate from model misspecification. We report the test for over-identifying restrictions (Sargen test) which reveals the validity of our instruments. The autocorrelation test also confirms the absence of serial correlation in the model. Finally, we also estimate a mean group estimator (Model 11) developed by Pesaran and Smith (1995) as another round of robustness tests. This model can be justified by the fact that the slope coefficient of the panel is likely to be heterogeneous due to the large time dimension (20 year period). It should be noted that the panel linear fixed effect, panel spline and GMM estimators all assume that the slope coefficient is poolable across the various cross-sections. However, with heterogeneous slope, the mean group estimator is well suited since it estimates the group-specific ordinary least squares regression and averages the estimated coefficient for the group in order to account for heterogeneity in the coefficient. Results of the MG estimator in Table 3 is also similar to the Panel Splines, fixed effects and GMM, with all pointing towards sustainability of the fiscal stance.

As another robustness check, we consider different values of the smoothing parameter ($\lambda$) used in the HP minimization criterion. We consider parameters by Backus and Kehoe (1992) who recommended $\lambda$ of 100^8^, Baxter and King (1999) suggested $\lambda$ value of 10, Correia et al. (1992) and Cooley and Ohanian (1991) suggest a value of 400. Finally, Ravn and Uhlig (2002) recommended a value of 6.5. We calculated GVAR and YVAR using these different $\lambda$ values for fixed effects and panel splines (see Table 9 in “Appendix”). The results reveal that using these different smoothing parameters does not change the results significantly. The debt reaction coefficient is positive and statistically significant in all the different specifications. It is not surprising since GVAR and YVAR serves as control variables in the model. Secondly, the reaction coefficients for both GVAR and YVAR are comparable across all the specifications. This proves the robustness of our model to different values of the HP smoothing parameter.

**Fig. 2:**
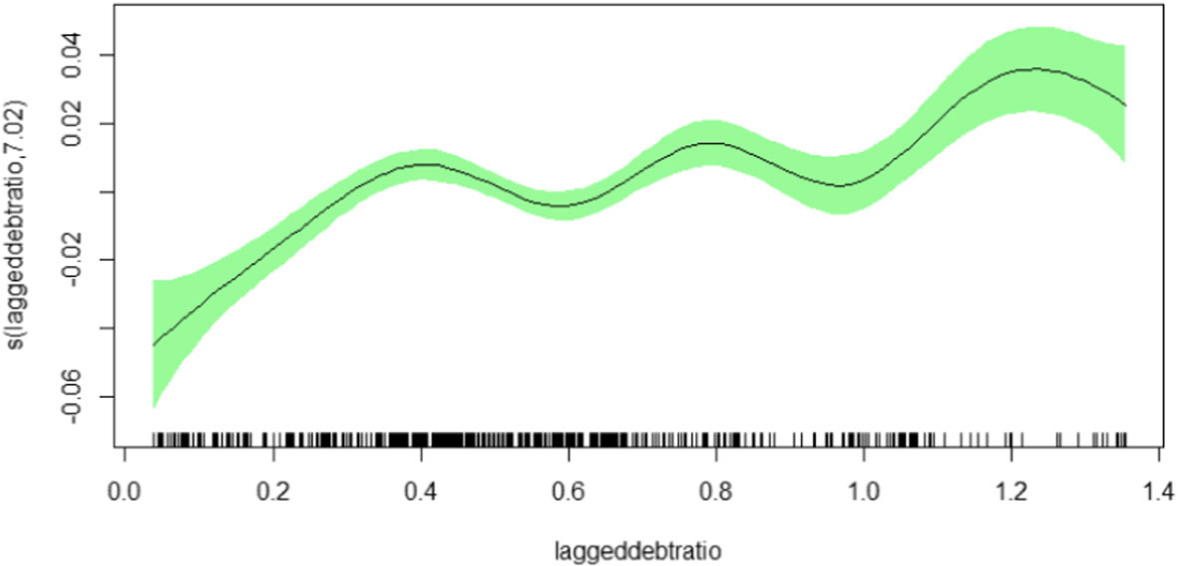
Full sample—without Greece. Smooth functions of the lagged debt-ratio for specification without Greece. Vertical axis depicts the estimated degree of freedom which governs the (non)linearity of the function

Next, we investigated the potential effect of each country in the panel datasets by excluding all the countries one after the other to ascertain the impact of the lagged debt-ratio on the primary balance. It turned out that the exclusion of Greece from the datasets yielded the most significant change in the reaction coefficient. Model 8 in Table 3 presents the estimation outcome for the full sample without Greece. It can be realized that the average reaction coefficient is clearly smaller now. This demonstrates that Greece had achieved very high primary surpluses, with the help of other EU economies, the Euro-zone and of the IMF, during its consolidation after its public debt crisis. The elimination of Greece from the sample also changes the shape of the smooth function, describing the reaction to higher debt ratios. Figure 2 demonstrates that the response to higher debt ratios rises with higher debt, but, not monotonously. Rather, there are oscillations indicating phases of ’fiscal fatigue’, meaning that the effort of counter-steering peters out and declines once the debt to GDP ratio exceeds certain thresholds.

All in all, our empirical analysis yields a statistically significant positive reaction coefficient on average and, thus, supports the view of sustainable debt policies in the countries under consideration. However, based on the spline estimations reveals that there is more information contained in the data. The smooth functions show that the response varies considerably across the distribution of the lagged debt-ratios. This effect is particularly pronounced for low and high values of public debt relative to GDP.

Figure 7 in “Appendix” depicts the residual diagnostics plot where we compare the residuals of the panel linear fixed effects model (specification 3 in Table 3) against the penalized spline model (specification 7 in Table 3). Since both models assume normally distributed residuals, it would be plausible to ascertain if this is indeed the case. The upper panel in Fig. 7 shows a quantile-quantile plot of the residuals for the two models. It can be observed that the panel spline residuals satisfy the normality assumption better than the linear fixed effects model since most of the residuals lie on the straight diagonal line between the theoretical quantile of a normal distribution and the sample quantile. Hence, the spline model performs better than the linear model. In the lower panel of Fig. 7, we plotted the fitted values against the residuals to explore the presence of heteroskedasticity. However, the residual plots for both models are similar and do not exhibit any pattern.

As a further robustness test, we estimated another semi-parametric model by way of a panel mixed effects model according to Ruppert et al. (2003), where the fixed effects represent the linear estimates and the random effects depict the nonlinearities. Results of the panel mixed effects model are shown in Table 7 in the “Appendix”. Comparing the panel mixed effects model to the panel spline fixed effects model (full model in Table 3) demonstrates that the estimates are almost the same and hence a confirmation for the robustness of our results.

### Results of subsamples

Due to the potential heterogeneity in the datasets, we explore the possibility of dividing the countries into clusters where each cluster is made up of countries with similar characteristics. We employ the K-mean approach (see Hartigan and Wong 1979) which entails finding clusters and cluster centers in a set of unlabeled data. Using this machine learning clustering technique, we choose tentatively the number of desired cluster centers and allow the K-mean algorithm to iteratively move the centers in a way as to minimize the sum within the variance of the cluster. Alternatively, an optimal number of clusters can be estimated by choosing the number of clusters for which the total within-cluster sum of square is minimized.

We estimate the clusters based on our variables of interest, notably the primary balance and the lagged debt-ratio. The results of the two cluster characteristics can be found in Table 6 in “Appendix”.^9^ Cluster 1 has an average debt of 105% and a primary surplus of 0.7% of GDP. Cluster 2, on the other hand, has a relatively smaller debt ratio of 42% and a primary surplus to GDP ratio of − 0.25%, i.e. a primary deficit.^10^ Cluster 1 is made up of mainly bigger economies in the EU namely: Belgium, Germany, Greece, France, Italy, Austria and Portugal. Countries that make up cluster 2 are as follows: Bulgaria, Czechia, Denmark, Estonia, Ireland, Spain, Cyprus, Latvia, Luxembourg, Hungary, Malta, Netherlands, Poland, Romania, Slovenia, Slovakia, Finland and Sweden.

Applying the penalized splines approach from above to these subsamples gives interesting results presented in Table 4. The first part (column two to four) considers the differences in the two clusters, the second part (column five and six) presents the results distinguished by crisis and non-crisis years.

**Table 4:** Clusters, crisis and non-crisis period

Variables	Panel Splines					GMM				Mean Group			
	Cluster 1	Cluster 1A	Cluster 2	Crisis	Non-crisis	Cluster 1	Cluster 2	Crisis	Non-crisis	Cluster 1	Cluster 2	Crisis	Non-crisis
*Parametric*													
Lagged debt-ratio	0.084***	0.025*	0.020**	0.076***	0.045***	0.057***	0.012*	0.017*	0.012*	0.052*	0.066***	− 0.149**	0.061**
	(0.015)	(0.013)	(0.009)	(0.017)	(0.007)	(0.035)	(0.016)	(0.010)	(0.006)	(0.031)	(0.024)	(0.06)	(0.029)
GVAR	− 0.0005***	− 0.0003**	− 0.003***	− 0.002***	− 0.0004	− 0.0003	− 0.0018*	− 0.0017**	− 0.0005**	− 0.002***	− 0.016**	− 0.021***	− 0.012**
	(0.000)	(0.000)	(0.000)	(0.0002)	(0.0002)	(0.0002)	(0.001)	(0.001)	(0.0002)	(0.001)	(0.006)	(0.007)	(0.005)
YVAR	− 0.00002	− 0.00003	0.0003***	− 0.00003	0.0001	0.000004	− 0.00006	− 0.0001	0.00003	0.0002	0.0004	− 0.005**	0.002
	(0.000)	(0.000)	(0.000)	(0.000)	(0.000)	(0.000)	(0.000)	(0.0002)	(0.000)	(0.0002)	( 0.001)	(0.003)	(0.002)
Inflation	− 0.125***	− 0.059***	− 0.044***	− 0.022	− 0.049***	− 0.057***	0.025	− 0.010	− 0.009**	− 0.075	− 0.037	0.343*	− 0.046
	(0.022)	(0.019)	(0.010)	(0.050)	(0.009)	(0.016)	(0.028)	(0.007)	(0.004)	(0.055)	(0.033)	(0.191)	(0.038)
Net export-ratio	0.300***	0.365***	0.173***	− 0.039	0.170***	0.330***	− 0.071	0.0167	0.033	− 0.0013	0.0211	− 0.377	0.013
	(0.093)	(0.074)	(0.031)	(0.069)	(0.029)	(0.072)	(0.091)	(0.053)	(0.028)	(0.073)	(0.075)	(0.261)	(0.065)
Lagged pbratio						0.315**	0.527***	0.481***	0.63***				
						(0.089)	(0.079)	(0.048)	(0.160)				
*Nonparametric*													
edf(lagged debt-ratio)	2.898***	4.676***	4.509***	3.507***	4.831***								
edf (GVAR)	4.976***	1.861*	8.166***	6.467***	3.936***								
edf (YVAR)	2.042	1.938	2.451**	2.150	1.977*								
edf (Net export-ratio)	2.342**	2.530***	5.905**	3.861**	5.818***								
Adj $R^{2}$	0.728	0.551	0.652	0.707	0.52					0.92	0.90	0.99	0.92
Observ	133	114	342	150	300	259	666	275	500	140	360	175	325
Sargen Test						7.0(0.9)	10.9(0.9)	18.4(0.9)	18.98(0.9)				
Autoc test (1)						− 18.59(0.000)	− 2.12(0.03)	− 1.94(0.05)	− 2.74(0.01)				
Autoc test (2)						0.55(0.58)	1.16(0.25)	0.61(0.54)	0.46 (0.65)				
Wald test of coefficients						2779.2(0.000)	153.8(0.000)	177.2(0.000)	280.93(0.000)				

Standard error are indicated in parenthesis. Panel splines estimations are based on Puetz and Kneib (2018). GMM estimations are based on Blundell and Bond (1998) estimator whereas the Mean Group estimations are based on Pesaran and Smith (1995)

*, ** and *** indicates statistical significance at 10%, 5% and 1% respectively

For all specifications, the results in Table 4 mainly support the general findings for the full sample from above. Active fiscal policy reacts in a positive manner to changes in debt, indicating sustainable policies. Higher spending exerts a negative effect on the primary balance and YVAR is statistically insignificant in most cases or positive but rather small for Cluster 2. Net export-ratio and Inflation are again statistically significant and positive and negative, respectively, except for the crisis years.

Looking closer at the estimation outcomes reveals some interesting insight. The upper part of Table 4 again presents the parametric part. For the cluster containing the bigger countries (Cluster 1) the debt coefficient turns out to be much larger compared to the smaller countries (Cluster 2) unless Greece is omitted. Cluster 1A in Table 4 gives the estimations with the omission of Greece from the sample. This yields a considerably lower estimate for the average reaction coefficient, just as in the case of the full sample, and the average reaction coefficient is only slightly larger than for Cluster 2 then. This shows once more that Greece had achieved large primary surpluses through its austerity programs during the crisis. Similarly, the negative inflation effect (our proxy for monetary policy) is stronger when Greece is included. The coefficient for net export-ratio is positive and significant, just as for the full sample.

Additionally, we split the data set in order to study the behavior of the reaction function in crisis periods and non-crisis periods. Regarding the crisis period, we choose the period between 2008 and 2014 that coincides with the global financial crisis which affected all EU countries.^11^ Further, in those years most of the EU countries were under the excessive deficit procedure due to the violation of the Maastricht Treaty criteria.^12^ Figure 8 in “Appendix” depicts the time series plot of the debt ratio to GDP ratios for selected European countries. Strong upward sloping debt ratios can be observed until 2014 (mostly beginning 2008), which begin to decline in 2014 in most of the countries. Therefore, the crisis period chosen is supported by the data.

As further justification for our chosen dates, we apply a structural break tests for each country where we consider the lagged debt-ratio ratio for each country. Identification of break is based on econometric structural break test according to Zeileis et al. (2003). The test is a combination of the F-statistics test by Andrews (1993) and Andrews and Ploberger (1994) to test for potential structural breaks and the algorithm by Bai and Perron (2002) to locate the optimal break dates series of the data. Break dates for each country have been reported in Table 8 in “Appendix”. It can be seen that most of the break dates for our lagged debt variable correspond to the year between 2008 and 2014. Hence, our structural break dates are justifiable.

Regarding the reaction function, the response of the primary surplus to public debt is stronger in crisis years than in non-crisis years which can be explained by austerity measures during crisis, while eventually less emphasis is put on debt sustainability in normal times. Moreover, the GVAR variable has a negative effect on the primary balance that is much stronger in crisis years compared to non-crisis years. Business cycles, YVAR, are statistically insignificant^13^ independent of whether crisis years or normal times are considered which may be due to the fact that we use the cyclically adjusted primary balance. Inflation and net export-ratio have the same effect as in the case of the full sample in non-crisis years, but, are statistically insignificant in crisis years. This may be due to unconventional policy measures during the crisis years, with interest rates near the lower bound among other things.

**Fig. 3:**
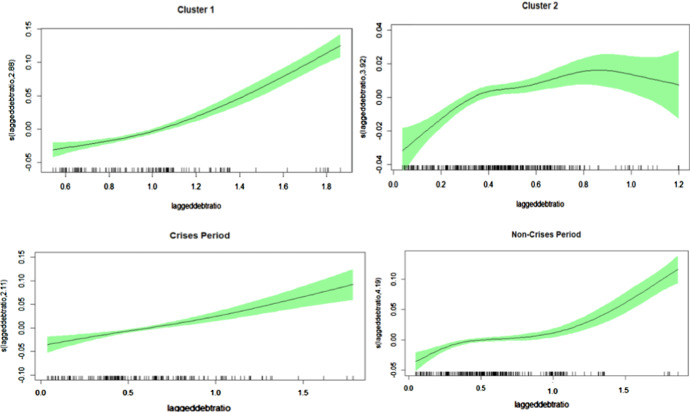
Smooth Functions. Smooth functions of the lagged debt-ratio for different clusters and (non) crisis period. Vertical axis depicts the estimated degree of freedom which governs the (non)linearity of the function

The smooth functions with estimated degrees of freedom higher than 1 demonstrate that the relationship between the primary surplus ratio and the explanatory variables is characterized by nonlinearities in most of the cases. Focusing on the public debt and primary balance to GDP ratio, Fig. 3 depicts the shape of the smooth function for our specifications Cluster 1 and Cluster 2 as well as for crisis and non-crisis periods.

Obviously, the response behavior is different in the two clusters: while the response in the case of Cluster 1 is almost monotonously increasing with higher public debt to GDP ratios, Cluster 2 with the smaller economies shows rather a flat curve that reminds somehow of a shallow inverse U-shaped form. For the small economies the response increases for low values of the debt ratio up to about 30%, then flattens until about 90% and for higher debt ratios the response decreases. This declining reaction of the primary balance for high values of the debt ratio is in line with the ’fiscal fatigue’ hypothesis as suggested in Ghosh et al. (2013), for instance. Comparing Crisis and Non-Crisis periods in Fig. 3 the latter supports the findings from above for the full sample, where the rise of the response becomes stronger for higher values of the debt ratio, while during crisis years there seems to be a constantly increasing reaction.

**Fig. 4:**
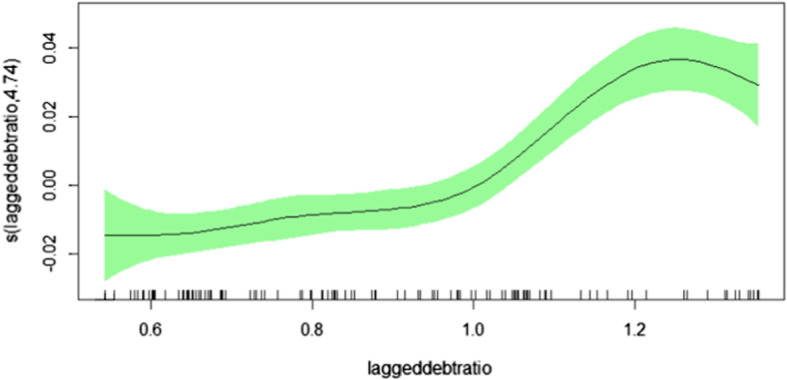
Cluster 1A—model without Greece. Smooth functions of the lagged debt-ratio for cluster 1A. Vertical axis depicts the estimated degree of freedom which governs the (non)linearity of the function

**Fig. 5:**
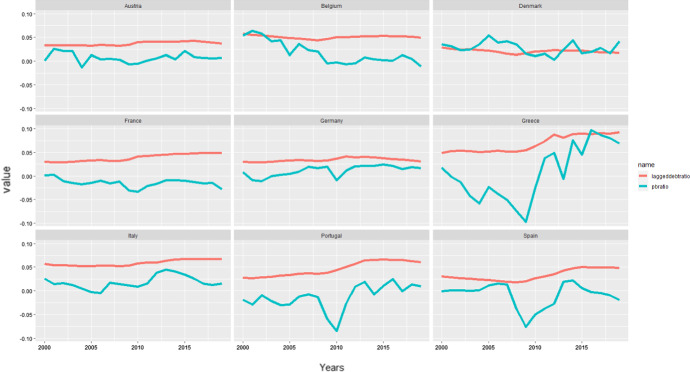
Primary balance and lagged debt-ratio for selected EU countries

**Fig. 7:**
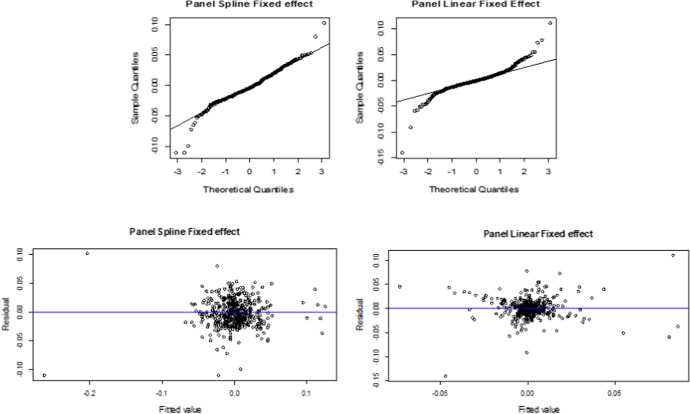
Residual diagnostic plots

**Fig. 8:**
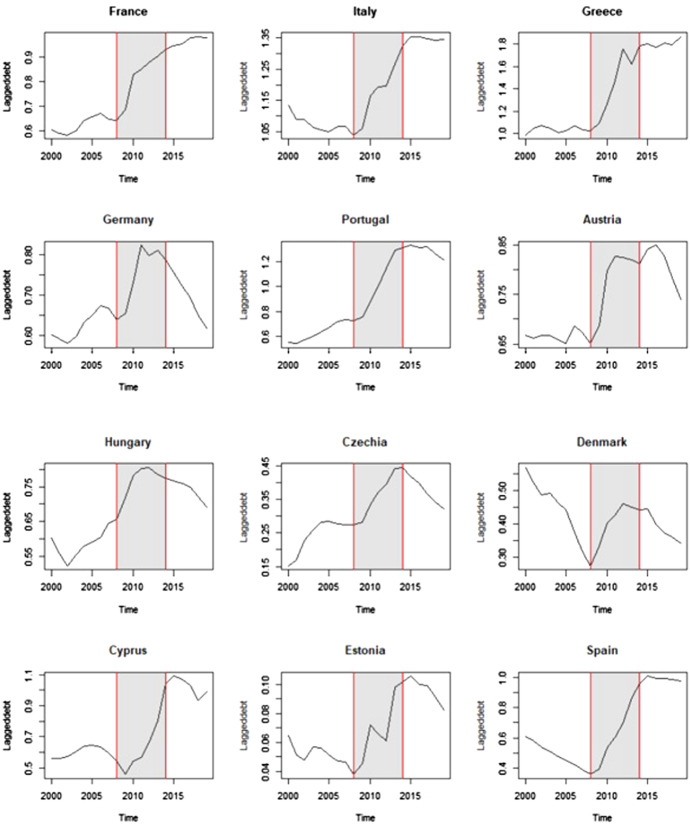
Lagged debt-ratio with shaded grey area (2008 and 2014) indicating crisis period

We should like to point out that the estimation for Cluster 1 without Greece, Cluster 1A in Table 4, yields a smooth function that again resembles a (left skewed) inverse U, implying a declining response for high debt to GDP ratios, as can be seen from Fig. 4. Hence, we see a tendency for ‘fiscal fatigue’ in those economies, just as for the ones of Cluster 2, when Greece is left out. This underlines once more that Greece had achieved very high primary surpluses in its process of fiscal consolidation after the beginning of the debt crisis.

Summing up our additional results, there are some interesting findings beyond the standard fixed effects and the full sample estimation. First, all specifications mainly support the full sample outcomes and, especially, reveal sustainable public debt policies. By distinguishing two (data-driven) clusters, it turns out that the fiscal response differs for each cluster. For the cluster with mostly small countries, in particular, ‘fiscal fatigue’ characteristics appear, meaning that the response to higher debt peters out and, finally, decreases as the debt to GDP ratios become larger. For the cluster containing the three largest economies, the response to higher debt is a monotonically increasing function of the debt to GDP ratio, thus, resembling the shape obtained for the full sample. However, once Greece is eliminated from that sample, that cluster reveals ‘fiscal fatigue’, implying that the response declines for high debt to GDP ratios in that cluster, too. Moreover, in non-crisis years there is some phase of easing for intermediate debt ratios, but during crisis years, the response is monotonously increasing, meaning a stronger reaction to higher debt if governments face large debt ratios.

Comparing these results with previous studies indicate some similarities despite the different approaches. For instance, Ghosh et al. (2013) with a different time span (1970–2007) and different sample choice (23 advanced economies, also outside Europe) and different empirical framework find the turning-point of a negative response at a debt ratio level of about 90–100%. Our estimations confirm these numbers, as for the small country subsample the response becomes negative at about 90% and for the group 1 without Greece (Cluster 1A) debt levels higher than 120% seem to indicate a negative reaction. Fournier and Fall (2015) also find fiscal fatigue behaviour (OECD economies, 1985:2013, threshold model) around a debt ratio of 170% of GDP and for the Euro area group (15 countries) with lower turning points (152% and 167%). Interestingly their results are also sensitive to the inclusion of Greece. Excluding Greece fiscal fatigue appears at a debt ratio around 120% of GDP—which is exactly in line with our results from above. On the other hand Checherita-Westphal and Zdarek (2017) find only week support for fiscal fatigue (18 Euro area) resorting to an approach taking actual fiscal behaviour into account.

## Conclusion

This paper has studied nonlinearities in a debt sustainability analysis by resorting to the penalized panel splines technique. Based on data for 25 EU economies over the years from 2000 to 2019, we estimated the fiscal response function by analyzing the relationship between the discretionary fiscal policy in terms of the cyclically adjusted primary balance to GDP ratio and the lagged debt to GDP ratio. A positive coefficient on average indicates a sustainable public debt policy, which is supported by our results for the full sample. However, we have seen that the relationship is not homogeneous across the distribution of the debt ratios, but, rather varies with the size of the debt ratio. It reveals strongly increasing reactions for small and high debt ratios, while it is rather flat for intermediate values. When Greece is eliminated from the sample, the response to higher debt ratios rises, but, not monotonously. Rather, there are phases of ‘fiscal fatigue’, meaning that the effort of counter-steering peters out and declines once the debt to GDP ratios exceed certain thresholds.

Furthermore, we have split the full sample into different categories to explore the heterogeneity in the data set. We classified the data set into two categories to ascertain the reaction function in times of debt crisis and in normal times. The reaction function in normal times exhibits a similar pattern as for the full sample, while during years of crisis the reaction to public debt has been found to be stronger. Additionally, we have used a data-driven algorithm to cluster the data set into two distinct groups. Results for the first cluster, consisting of mainly larger EU economies, shows a strong response of the primary balance to increases in the debt ratio yielding strong evidence for fiscal sustainability as long as Greece is included in that sample. Once Greece is taken out, however, the average reaction coefficient takes on a much lower value and the smooth function is characterized by an inverted U-shaped relationship: it rises with increasing debt to GDP ratios, reaches a maximum and, then, declines again. That shape was found for the second cluster, too, which consists of mostly smaller EU economies. To test the robustness of our results, we have estimated a fiscal reaction function using spline mixed effects models for our panel data set and the main results remain unchanged, pointing out the robustness of our estimations.

These refinements are particularly important for policy implications and help to improve fiscal recommendations as they indicate that the status of the current debt and the economic situation is essential for the assessment and evaluation of sustainability. Our results show that, yes, size does matter! The level of the explanatory variable influences the reaction coefficient and, thus, fiscal sustainability. This means policy recommendations need to consider the status of the current debt situation in order to be successful, as the reaction shows different behavior for low debt levels compared to medium or high ratios. Our findings are in line with other literature contributions that focused on nonlinear fiscal behaviour and study “fiscal fatigue”. This also holds true for the special case of Greece.

As a potential limitation, it must be noted that the estimated semi-parametric model does not take into account the issue of cross-sectional dependence. Since we are dealing with countries that are heavily linked together economically, there is the possibility that the residuals would also show cross-sectional dependence, which could affect the consistency of the estimates. None of the estimators used in this paper account for cross-sectional dependence. Hence it will be plausible for future research to consider semi-parametric models which accounts for cross sectional dependence in the panel data context. Also, our data set covers observations until 2019 only. Certainly, the effects of the pandemic requiring immediate unprecedented policy responses by governments and leading debt ratios to extraordinary levels are not covered yet. All these limitations are open for further research.

## Figures and Tables

**Fig. 6:**
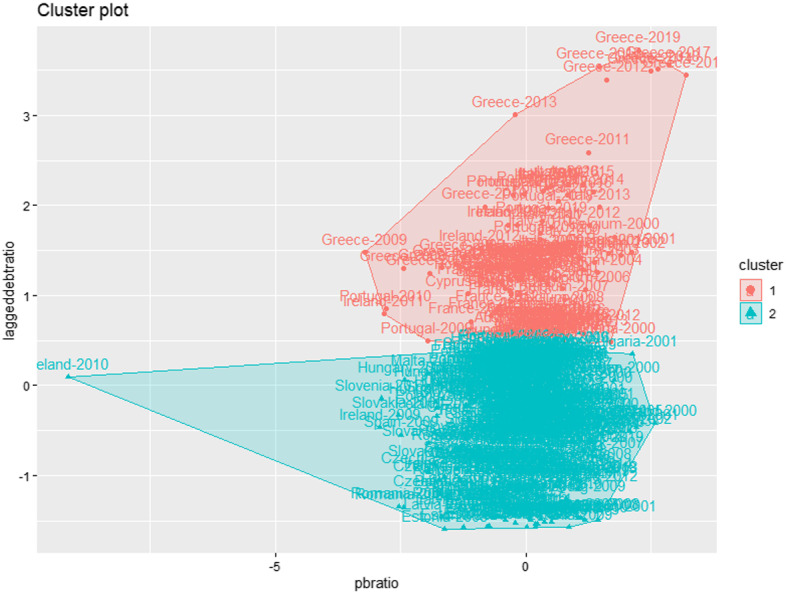
K-mean cluster plot

## Appendix

See Figs. 5, 6, 7, 8 and Tables 5, 6, 7, 8, 9.

**Table 5:** Correlation matrix

	Pbratio	Debt	GVAR	Inflation	YVAR	Net export-ratio
Pbratio	1					
Lagged debt-ratio	0.212	1				
GVAR	− 0.380	− 0.057	1			
Inflation	− 0.001	0.302	0.043	1		
YVAR	− 0.103	− 0.120	0.118	0.110	1	
Net export-ratio	0.264	− 0.097	− 0.041	0.254	− 0.609	1

**Table 6:** Clustering characteristics, measured in % of GDP

Cluster	Lagged debt-ratio	Pbratio
1	105	0.7
2	42.2	− 0.25

**Table 7:** Robustness test—spline mixed effect model

Variables	Response variable: Pbratio			
	1	2	3	4
*Parametric*				
Lagged debt-ratio	0.056***	0.066***	0.054***	0.058***
	(0.001)	(0.007)	(0.007)	(0.007)
GVAR	− 0.002***	− 0.002**	− 0.002***	0.048***
	(0.000)	(0.000)	(0.000)	(0.000)
YVAR	0.0001	− 0.000002	− 0.0001	− 0.003
	(0.000)	(0.000)	(0.000)	(0.000)
Inflation		− 0.027**	0.049***	− 0.056***
		(0.008)	(0.001)	(0.007)
Net export-ratio			0.145***	− 0.001***
			(0.000)	(0.000)
*Nonparametric*				
edf(lagged debt-ratio)	6.462***	7.083***	6.828***	7.206***
edf (GVAR)				8.510***
edf (YVAR)				2.909*
edf (Net export-ratio)				6.881***
Adj $R^{2}$	0.174	0.156	0.30	0.471
Observ	500	500	500	500

Standard error are indicated in parenthesis

*, ** and *** indicates statistical significance at 10%, 5% and 1% respectively

**Table 8:** Structural break test based on Zeileis et al. (2003)—debt to GDP ratio

Countries	Optimal number of breaks	Break dates
Austria	2	2009, 2016
Belgium	3	2003, 2006, 2009
Bulgaria	3	2002, 2005, 2015
Cyprus	2	2010, 2013
Czechia	4	2002, 2009, 2012, 2016
Denmark	4	2002, 2005, 2009,2015
Estonia	4	2005, 2009, 2012, 2016
Finland	4	2006, 2009, 2012, 2015
France	4	2003, 2009, 2012, 2015
Germany	3	2003, 2009, 2016
Greece	2	2010, 2013
Hungary	3	2006, 2009, 2016
Ireland	3	2008, 2011, 2015
Italy	3	2002, 2009, 2013
Latvia	2	2009, 2015
Luxembourg	2	2008, 2012
Malta	3	2003, 2006, 2015
Netherlands	4	2005, 2008, 2012, 2016
Poland	3	2003, 2010, 2014
Portugal	3	2004, 2009, 2012
Romania	2	2004, 2010
Slovakia	4	2002, 2005, 2009, 2012
Slovenia	2	2010, 2013
Spain	3	2003, 2009, 2012
Sweden	3	2002, 2006, 2014

**Table 9:** Robustness test—varying the HP smoothing parameter

Variables	Panel fixed effects					Panel penalized splines			
	$\gamma$ = 100	$\gamma$ = 400	$\gamma$ = 10	$\gamma$ = 6.5		$\gamma$ = 100	$\gamma$ = 400	$\gamma$ = 10	$\gamma$ = 6.5
*Parametric*									
Lagged debt-ratio	0.078***	0.078***	0.087***	0.089***		0.042***	0.044***	0.042***	0.042***
	(0.017)	(0.018)	(0.017)	(0.017)		(0.007)	(0.007)	(0.007)	(0.007)
GVAR	− 0.002***	− 0.002***	− 0.002***	− 0.002***		− 0.022***	− 0.001***	− 0.002***	− 0.002***
	(0.0001)	(0.0001)	(0.0002)	(0.0001)		(0.000)	(0.0001)	(0.0002)	(0.0002)
YVAR	− 0.0001*	− 0.0001	− 0.0002*	− 0.0002*		0.001	0.0002***	− 0.0001	− 0.0001
	(0.000)	(0.000)	(0.000)	(0.000)		(0.000)	(0.000)	(0.000)	(0.000)
Inflation	0.042	0.038	0.045	0.045		− 0.049***	− 0.051***	− 0.051***	− 0.051***
	(0.035)	(0.036)	(0.035)	(0.035)		( 0.009)	(0.010)	(0.010)	(0.010)
Net export-ratio	− 0.024	− 0.024	− 0.014	− 0.013		0.172**	0.182***	0.190***	0.191***
	(0.039)	(0.040)	(0.039)	(0.039)		(0.030)	(0.030)	(0.030)	(0.030)
*Nonparametric*									
edf(lagged debt-ratio)						5.806***	5.581***	5.695***	5.706***
edf(GVAR)						6.995***	5.990***	6.822***	6.783***
edf(YVAR)						2.987**	3.225**	2.001	1.762
edf(Net export-ratio)						6.360***	6.480***	6.257***	6.258***
Adj $R^{2}$	0.292	0.273	0.288	0.288		0.609	0.552	0.603	0.601
Observ	475	475	475	475		475	475	475	475

Standard error are indicated in parenthesis

*, ** and *** indicates statistical significance at 10%, 5% and 1%, respectively

## Footnotes

1 See Puetz and Kneib (2018) for further details.

2 For Inflation this turned out to be the case, so it has been included in a linear manner. The effective degree of freedom is a meaningful and scale-free measure of the complexity of the penalized spline fit (Harezlak et al. 2018).

3 Fiscal policy is one of the most powerful instruments of a government to achieve political goals. The primary balance reflects the quantitative outcome of discretionary tax and spending decisions, or rather of the policy design, respectively.

4 According to the tax smoothing principle, the public deficit should be used such that the tax rate is constant over time to minimize the excess burden of taxation.

5 We used the GDP price deflator as a proxy for inflation with 2015 the base year.

6 The standard errors were rounded to the third and fourth decimal place, respectively.

7 As mentioned above, Inflation exerts a linear effect, thus, no smooth function is needed. Hence, the model is rather a semi-parametric one.

8 We used HP smoothing parameter of 100 to calculate the GVAR and YVAR for all the original specifications.

9 We estimated the optimal number of clusters which turned out to be 4. However, using 4 clusters implies less number of observations for each group. Given that penalized splines require quite a number of observations for efficient estimation, we use 2 clusters in order to avoid loss in degrees of freedom.

10 A graphical view of the clusters plot is shown by Fig. 6 in “Appendix”.

11 Additional estimations show that the results are quite robust with regard to the cut in 2014, they do not differ much if the time span for the crisis is slightly extended or shortened.

12 See European Commission (2021) for information as regards the excessive deficit procedure.

13 Again, the standard errors are rounded to the third and forth decimal place, respectively, in Table 4.
